# Genetic characterization of worldwide *Prunus domestica* (plum) germplasm using sequence-based genotyping

**DOI:** 10.1038/s41438-018-0090-6

**Published:** 2019-01-01

**Authors:** Tetyana Zhebentyayeva, Vijay Shankar, Ralph Scorza, Ann Callahan, Michel Ravelonandro, Sarah Castro, Theodore DeJong, Christopher A. Saski, Chris Dardick

**Affiliations:** 1 The Schatz Center for Tree Molecular Genetics, Department of Ecosystem Sciences and Management, The Pennsylvania State University, University Park, PA, 16802 USA; 2 Genomics and Computational Biology Laboratory, Clemson University, Clemson, SC 29634 USA; 3 USDA Appalachian Fruit Research Laboratory, Kearneysville, WV 25430 USA; 4 UMR BFP1332 - INRA-Bordeaux, Bordeaux II University, 33882 Villenave d’Ornon, France; 5 Department of Plant Sciences, University of California, Davis, CA 95616 USA

**Keywords:** Plant genetics, Plant breeding

## Abstract

*Prunus domestica* commonly known as European plum is a hexaploid fruit tree species cultivated around the world. Locally it is used for fresh consumption, in jams or jellies, and the production of spirits while commercially the fruit is primarily sold dried (prunes). Despite its agricultural importance and long history of cultivation, many questions remain about the origin of this species, the relationships among its many pomological types, and its underlying genetics. Here, we used a sequence-based genotyping approach to characterize worldwide plum germplasm including the potential progenitor Eurasian plum species. Analysis of 405 DNA samples established a set of four clades consistent with the pomological groups Greengages, Mirabelles, European plums, and d’Agen (French) prune plums. A number of cultivars from each clade were identified as likely clonal selections, particularly among the “French” type prune germplasm that is widely cultivated today. Overall, there was relatively low genetic diversity across all cultivated plums suggesting they have been largely inbred and/or derived from a limited number of founders. The results agree with *P. domestica* having originated as an interspecific hybrid of a diploid *P. cerasifera* and a tetraploid *P. spinosa* that itself may have been an interspecific hybrid of *P. cerasifera* and an unknown Eurasian plum species. The low genetic diversity and lack of true wild-types coupled with the known cultivation history of Eurasian plums imply that *P. domestica* may have been a product of inter-specific cross breeding and artificial selection by early agrarian Eurasian societies.

## Plums: DNA reveals evolutionary history of European plum varieties

The plums grown today for dried prunes likely originated from hybrid crosses and artificial selection by early agrarian societies, a genetic analysis shows. Chris Dardick from the US Department of Agriculture’s Appalachian Fruit Research Laboratory in Kearneysville, West Virginia, and coworkers sequenced more than 100,000 single DNA letters scattered across the genomes of 405 different samples of the European plum (*Prunus domestica*). The plants clustered genetically into four groups that corresponded with known plum varieties, such as greengages and mirabelles, but not with others, including damsons. Overall, the cultivated plums harbored a low level of genetic diversity, suggestive of repeated inbreeding from a small number of founder plants. The data also point to the European plum originating from a hybrid cross between the cherry plum (*Prunus cerasifera*) and blackthorn (*Prunus spinosa*).

## Introduction

European plum *Prunus domestica* L. is a polymorphic allopolyploid (hexaploid) species (2*n* = 6*x* = 48) commercially grown worldwide for a variety of uses including fresh fruit, prunes, distilling, and as processed additive ingredients. This plum species, commonly referred to as “European plums” or “prune plums”, is distinct from the large round diploid “Japanese plums” (*Prunus salicina*) which are widely grown for fresh market consumption. *P. domestica* plums are critical components of human diets as prunes have been shown to have a broad range of health promoting activities including protection against cardiovascular disease, diabetes, digestive disorders, and osteoporosis^1,2^. Historical evidence suggests *P. domestica* and other Eurasian plum species including *Prunus cerasifera* (Cherry plum) and *Prunus spinosa* (“Sloe” or “Blackthorn” plum) were important to the development of early European societies. Stones from all three species have been uncovered from Neolithic archeological sites dating back to 4000–6000 BC in Germany and the Ukraine^3^.

The plum or “prumnon” was first recorded in Archilochus’s “Pollux” written in the 7th century BC^3^. Around the birth of Christianity, the Roman author Pliny gave the first clear account of “Damascus plum” where he described a wide variety of cultivars with different fruit types and colors, their uses, and grafting onto “sorb” rootstocks (probably *Prunus cerasifera* or *Prunus spinosa*), which is still a common practice today. Roman depictions of plum fruit provide further evidence that at least some of the *P. domestica* varieties grown at that time were very similar or even possibly the same as those still grown today. References to different types of plums being imported from Syria or Persia and the fact that plums were not native to some of the Neolithic sites indicates that they were under human selection and cultivation in the Caucasus region long before their introduction into Europe. The propensity of *P. domestica* to form root suckers would have provided a simple and efficient way to distribute clonal material across long distances even before grafting techniques had been developed.

*P. domestica* is thought to have originated from the Middle East, specifically, in the area south of the Caucasus between the Black Sea and the Caspian Sea that encompasses Georgia, Armenia, Azerbaijan, and the northern plateau of Iran^4^. Vavilov^5^ placed the center of its origin in the region south of Caucasian Mountains through the Caspian Sea, in the area overlapping the distribution of *P. cerasifera* L. and *P. spinosa* L; which were likewise concurrently distributed across Europe for cultivation, consumption, and use as rootstocks. Widespread feral growth, the apparent lack of natural stands in forests, and human movement of both cultivated and wild type plums across the continent has made determining the origin of *P. domestica* extremely difficult. The existence of wild forms of *P. domestica* in the Caucasus region was recurrently reported in the 1930s based on morphological traits but failed validation from cytogenetic analyses^5,6^. Recent discovery of wild *P. domestica* stands in Xinjiang province in northwestern China prompted the hypothesis that European plum may be of Chinese origin^7,8^. However, narrow molecular diversity of botanical material from Xinjiang found that the Chinese *P. domestica* was likely feral resulting from escape and naturalization^9^.

**Fig. 1:**
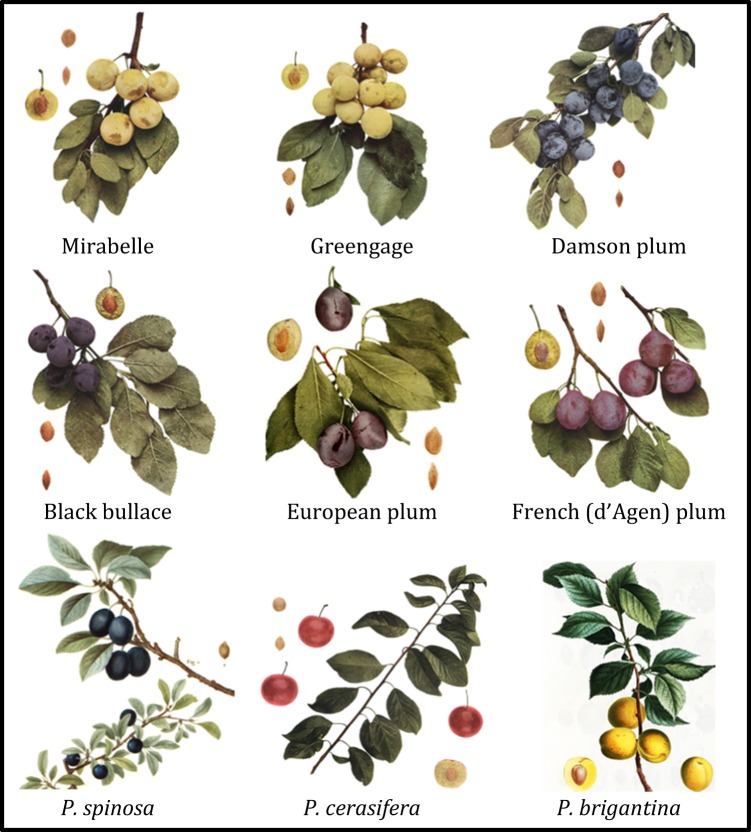
Fruit-type diversity of *P. domestica* and potential ancestral species. Illustrations were reprinted from Hedrick^4^ with the exception of *P. spinosa* and *P. brigantina*. These early 19th century illustrations were created by the artist Pancrace Bessa and published in “Treats the trees and shrubs grown in France in the open ground by [H.L.] Duhamel [du Monceau; ed. by Etienne Michel (vol 1–4) and J.L.A. Loiseleur-Deslongchamps (volumes 5–7)]

Consequently, the origin of *P. domestica* has remained a matter of debate for nearly a century. A major complication is the wide range of intraspecific variations and transitional forms^10^. Traditionally, plum cultivars have been divided into a number of different pomological groups; small fruited mirabelle plums, damsons, small wild plums or bullaces, greengages, prune plums, and large-fruited European plums (Fig. 1)^11–13^. However, clear delineations between these groups are extremely difficult to define due to the inherent phenotypic variability across the germplasm^3,4,13^.

Molecular phylogenetic analyses have more recently confirmed the close relationships among *P. cerasifera*, *P. insititia*, *P. domestica* L., *P. spinosa* L., and the “Marmot” plum *Prunus brigantina* and dated their divergence from a common Eurasian ancestor to the Oligocene (31Myr)^14^. It is now generally accepted that *P. domestica* is most likely an interspecific hybrid of *P. cerasifera* and *P. spinosa*. However, defining the precise genetic history of these events has been complicated by the fact that *P. cerasifera* and *P. spinosa* are found as diploid and polyploid individuals, sometimes having morphologies very similar to *P. domestica*. In fact, hybridization experiments and restriction site analysis of Ribosomal RNA genes indicated that *P. spinosa* itself may be an inter-specific hybrid having *P. cerasifera* as one of the parents^15–19^. Crane and Lawrence^20^ and Rybin^6^ performed direct interspecific hybridization experiments between *P. cerasifera (2x)*, and *P. spinosa (4x)* and found that, while the vast majority of the resulting offspring were infertile, a very small number of fertile hybrids that were morphologically similar to *P. domestica* could be obtained^6^. Still, many questions remain unresolved as to the origins of the various pomological plum groups and whether they represent independent inter-specific hybridization events or arose from the same or limited number of events^3,6,12,13,20^.

More recently, genetic diversity of Eurasian plum varieties and species has been studied using bi-parentally transmitted nuclear and maternal cytoplasmic (chloroplast, mitochondria) markers. Microsatellite (SSR) and Inter-simple Sequence Repeat (ISSR) markers were used for characterization of *P. domestica* germplasm across Europe in several geographically distant countries^21–24^, in Turkey^25^ and China^9,26^. This approach enabled a reconstruction of parental lineages among related European plum cultivars^27^ and molecular characterization of “Reine Claude” types in Spain^28^. Using the allelic variants of the S-RNase gene, groups of cross-compatible and incompatible cultivars were generated for Latvian plums^29^, and selected cultivars propagated in Hungary and Slovakia^30,31^. In several studies cytoplasmic (chloroplast) and nuclear SSR markers were used to reveal genetic diversification within *P. domestica* and to attempt to resolve its origin. Based on chloroplast markers it was shown that *P. cerasifera* was a likely progenitor, at least in the maternal lineage^21,32^. Two main cpDNA haplotypes were identified, however, major haplotypes were not clearly associated with pomological groups^21^. Nuclear DNA markers, on the other hand, indicated a potential contribution of *P. spinosa* to the *P. domestica* genome^14,21^. Unfortunately, the limited number of molecular markers used for characterization of plum germplasm to date, have not yet provided a clear picture of the genetic relationships among Eurasian plum germplasms.

Restriction site-associated sequencing has been an effective method for identifying and screening high-resolution polymorphisms within and between populations, ecotypes, and species^33,34^. In the present study, we generated a set of sequence-based SNP markers densely distributed across the *Prunus* genome in order to: (1) investigate genetic relationships among cultivated plums from different pomological groups that originated under the influence of different ecological factors and were propagated in different geographical regions; (2) estimate the extent of variation among *P. domestica* germplasm and identify diagnostic molecular markers; and (3) estimate the potential contributions of other plum species *P. cerasifera* and *P. spinosa* to the nuclear genome of hexaploid *P. domestica*. The resulting phylogenetic relationships along with our understanding of the origins, history, and future of plum cultivation are discussed.

## Results

### SNP discovery

In total, we sequenced 405 DNA samples representing different pomological groups of *P. domestica* and other Eurasian *Prunus* species including the potential *P. domestica* progenitors *P. cerasifera* and *P. spinosa* (Table 1, Table S1).

**Table 1:** Plum pomological groups and species in this study

Plum group/species	Code	Accessions
European plums	EUR	195
Greengages	GRG	46
Mirabelles	MIR	15
Prunes	DAP	107
*P. insititia*	Pi	9
*P. spinosa*	Psp	20
*P. cerasifera*	Pc	10
*P. brigantina*	Pbr	2^a^
*P. simonii*	Psi	1
Total		405

^a^Including *P. brigantina* × *P. cerasifera* hybrid

**Fig. 2:**
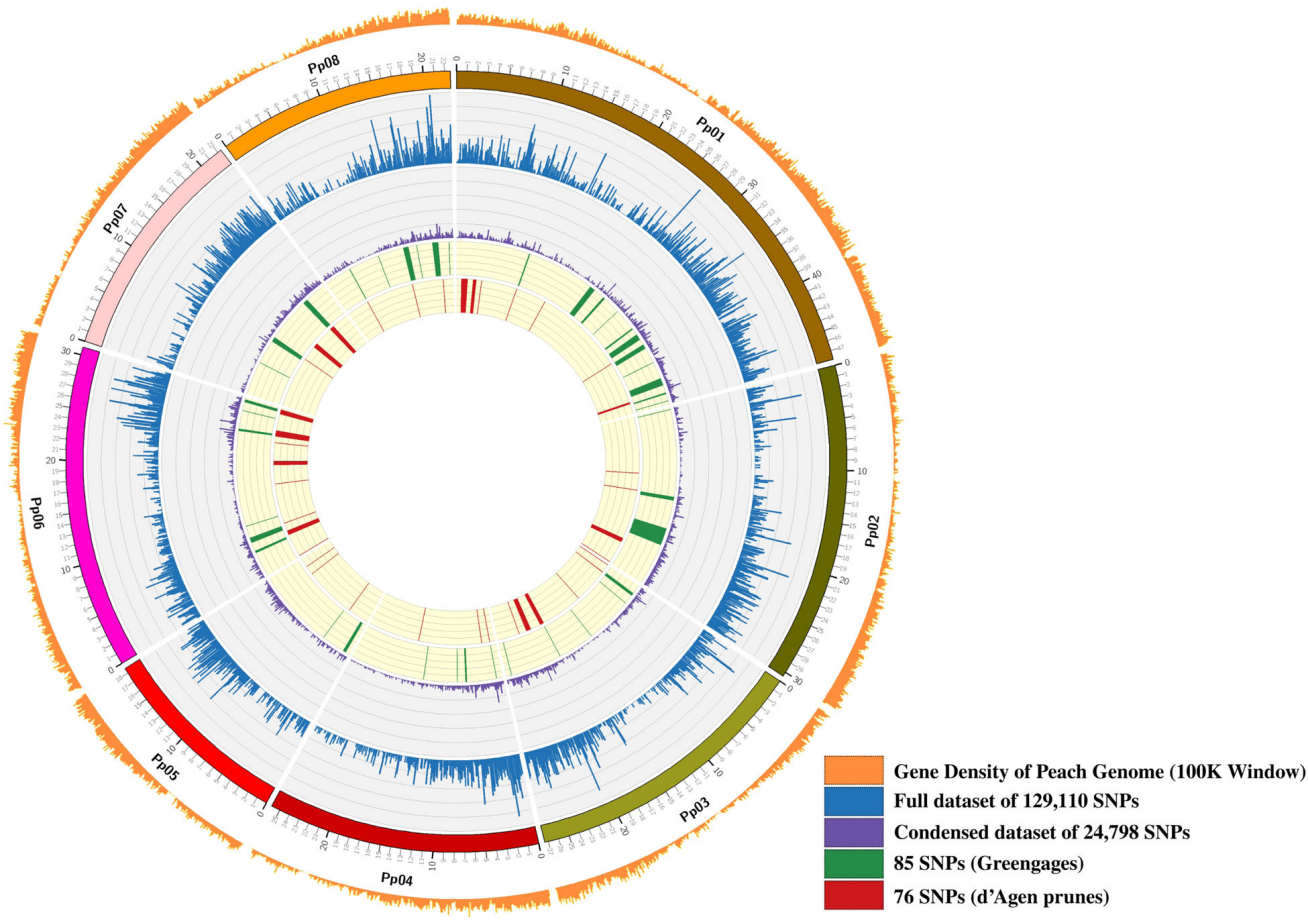
Genome-wide distribution of SNPs generated for genetic analyses of 405 accessions. The number of genes in the peach reference genome and the SNPs analyzed in the datasets were calculated per 100 kb genomic intervals. Peach pseudochromosomes are labeled as Pp01 to Pp08 and shown in different colors. Scale of pseudochromosome sizes is in Mb. A small subset of SNPs that were found to be differentiating between Greengage (green) and d’Agen plum (red) types are also shown

Five *Pst*1-digested genomic library pools were sequenced across five Illumina HiSeq2500 lanes producing 2051 million (mln) raw reads. Of these, 97.8% (2005 mln) reads were retained after filtering for base call accuracy, barcode match, and presence of the *Pst1*-restriction site (CTGCA/G). After demultiplexing, the average numbers of reads per DNA sample was 4.93 million reads. Only 5.6% (23 out 405 accessions) had fewer than 1 mln reads. All sequences were aligned against the *P. persica* genome assembly v2.1 for SNP identification. The average depth of stacks for variant calling was 221 and varied from 6 to 778. A total of 178,403 unfiltered sites were identified including monomorphic SNPs across all plum accessions. Filtering for SNPs present in more than 80% of accessions resulted in a dataset of 129,110 SNPs that were evenly distributed throughout the *P. persica* pseudochromosomes with little or no large-scale bias (Fig. 2). To keep uncommon variants that are specific to diploid *Prunus* species and potentially contributing to the hexaploid genome, we omitted two common filters, minor allelic frequencies and missing individual rate.

A total of 400 sequence variants derived from the chloroplast (cp) genome were also identified. Of these, 14 SNPs were present in more than 80% of accessions (Table S2). After filtering for Minor Allelic Frequencies (MAF) > 0.05, the only SNP with allelic frequency of 0.324 was a synonymous C/T substitution at 57,543 nt (+) within the large subunit of ribulose 1,5-bisphosphate carboxylase/oxygenase (rbcL) gene.

### Genetic diversity and phylogenetic analysis

**Fig. 3:**
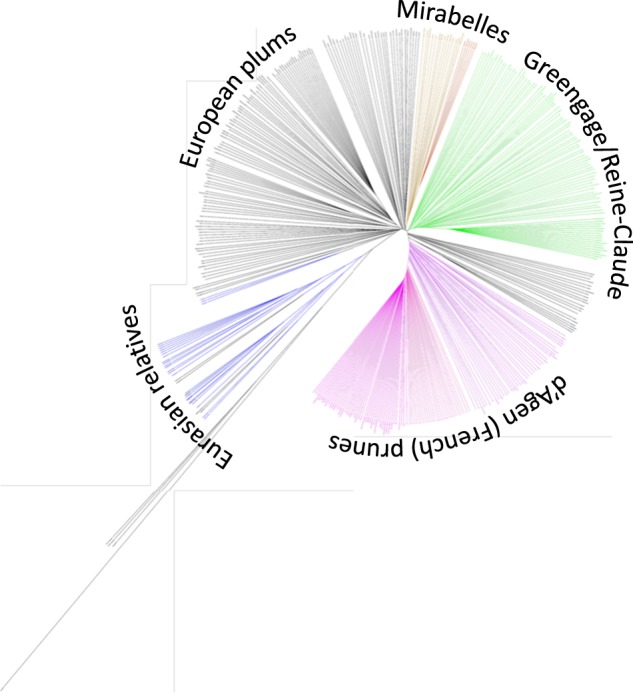
Phylogeny of *P. domestica* and related species. Dendrogram was generated using UPGMA clustering method on dissimilarity matrix computed for 405 accessions and 103,382 nuclear markers. A high-resolution version of the dendrogram is available as Figure S1. Branches are shown in ascending order based on pairwise distances. Different *Prunus* species and pomological groups are labeled and defined by colors—*P. cerasifera* and tetraploid *P. spinosa* (blue), European plums (black), mirabelles (brown), greengages (green) an d’Agen prunes (fuchsia). More intensive colors are shown for varieties with morphological traits that are more typical for the pomological group and likely represent the “core” germplasm

We investigated genetic relationships among all 405 plum accessions by performing a hierarchical clustering analysis (between-group average linkage method, or UPGMA) on a dissimilarity matrix calculated in R. In total, 103,382 polymorphic SNPs out of 129,110 sites exported from Stacks were used for phylogenetic classification (Fig. 3; Figure S1). The biological and technical replicates included in the inter-plate control and analysis were used to assess dendrogram quality. Results showed robust and consistent clustering of identical samples and an overall absence of a strong Illumina lane or geographic location effect on genetic signals.

### Prunus domestica germplasm structure

Distinct clades among domesticated hexaploid plums reflected some, but not all, of the known pomological groups including European plums (EUP), Mirabelles (MIR), Greengages (GRG), and d’Agen prune plums (DAP) (Fig. 3; Figure S1; Table S3). Differentiation of other pomological groups including *P. insititia*, “Damsons”, and “Bullaces” was not supported as such identified individuals did not form discrete clades. Among the four identified clades, subclades consisting of technical replicates, biological replicates and/or known clonal varieties could be distinguished in all pomological groups. With very few exceptions, varieties with known parentage clustered as expected within clades supporting the accuracy of the phylogenetic classification (Table S1). Using the “collapse subtree option” in Geneious^35^ we estimated a cutoff value for discriminating groups of clonal material using the genetic distances among DAP germplasm including samples from known clones, technical replicates, and seedlings. A subclade comprising 34 individual trees derived from self-pollination of the commercial variety “Improved French” and a subclade of 46 samples that included known DAP clones and technical replicates collapsed at the thresholds of branch lengths 0.392 and 0.373, respectively, while advanced generations of hybrids having d’Agen in their pedigree collapsed at a threshold of 0.454. Consequently, a conservative branch length cutoff value of 0.363 was chosen to distinguish clones across the rest of the dataset. This resulted in the identification of 16 clonal groups that were distributed in all four clades. These results are consistent with the known history of plum domestication as numerous cultivars have been selected by propagation of sports or suckers spontaneously showing new characteristics. It is important to note that this analysis may not have captured all clones as a handful of samples that are likely to be clonal did not meet the threshold (Table S1).

Due to the large number of samples used in this study, a detailed analysis of individual cultivars and their genetic relationships is not reported here. General results from the four distinguishable *P. domestica* clades are given below.

#### European plums (EUP)

European plums include many old English and Eastern European cultivars. Members of this group also show a high degree of morphological diversity ranging from small round bullaces to large oblong plums. The EUP clade showed the highest level of genetic diversity. Nine clonal clades were identified among EUP types. The largest consisted predominantly of “Pozegaca” or “Quetsche” types which are round, small fruited plums commonly referred to as “German prunes”. These are believed to be ancient cultivars that are still preferred in Eastern Europe but have largely been pushed out of production due to the plum pox virus epidemic^36^. Other smaller clonal clades consisted of varieties of French origin with mixed but often related names and the so-called “Italian” plums which produce large, oblong shaped fruit (Table S1)^37^. A set of EUP cultivars with diverse origins including “Ruth Gerstetter”, “Topfirst”, “Perdrigonne” and “Cacanska Rana” formed a more distant and poorly defined EUP subclade. “Ruth Gerstetter” is recorded to have been a cross between “Bonne de bry” and “Czar”. Our results show that “Bonne de bry” is likely a Greengage type. The other parent, “Czar”, was not included in this study, however, other plums with “Czar” parentage including “Edda” and “Herman” fall within the same clade. Likewise, “Topfirst” has “Ruth Gerstetter” as a parent and also falls within this group. One possibility is that this EUP subclade is comprised of plums with mixed heritage.

#### Mirabelles (MIR)

Mirabelle plums are small, typically red or yellow in color, and slightly oval in shape. They are often found growing feral and most commonly prepared as jams or jellies. The MIR clade represented a very narrow range of germplasm that was not far removed from EUP plums. Three small clonal groups consisting of 3–6 varieties each were identified (Table S1).

#### Greengages

The name “Greengage” comes from Sir William Gage who supposedly imported these small, often green-fruited types from France in the early 1700s^38^. Many of the cultivars carry their French name “Reine Claude” in honor of Queen Claude who ruled France in the early 1500s. Three groups of clonal material were identified with a single large group (18 members) consisting of cultivars named as “Reine Claude” types. These findings suggest that a significant portion of the commercial “Greengage” germplasm is clonal.

#### D’Agen prune plums (DAP)

DAP is the most economically important group of plums and includes the vast majority of commercially growth prunes. The DAP clade (144 accessions) was composed of commercial d’Agen prunes from different geographical regions, progeny from self-pollination of the d’Agen prune “Improved French”, cultivars with known d’Agen types in their pedigree and a few unclassified cultivars. Nearly all individuals named as d’Agen types were identified as clones, with the exception of three d’Ente varieties collected from INRA that fell into the EUP clade and d’Ente-jaune which is likely a self-pollinated d’Agen seedling (Table S1). These results indicate that worldwide commercial prune production is predominantly a monoculture.

### Eurasian (EUR) plums and *P. domestica* ancestry

Several diploid plum species were examined including accessions of *P. simonii* (native to China), the interspecific hybrid rootstock *P. cerasifera* × *P. munsoniana* (‘Marianna 2634’) (note: *P. munsoniana* is native to North America), several diploid *P. cerasifera* selections, *P. brigantina*, and a hybrid of *P. cerasifera* × *P. brigantina*. Tetraploid *P. spinosa* accessions were collected from the U.S., Sweden, and Portugal. Accessions recorded as *P. insititia* included the rootstocks “Saint Julien” as well as several varieties collected from INRA.

The non-Eurasion plum *P. simonii* formed a distinct out-group and, to a lesser extent, so did the *P. cerasifera* × *P. munsoniana* hybrid “Marianna 2624”. In contrast, all *P. cerasifera*, *P. spinosa*, and *P. brigantina* selections displayed relatively short genetic distances to *P. domestica*. These species did not form discrete clades and three potentially clonal groups were identified (Table S1). These include a group of *P. cerasifera* accessions from the UC Davis germplasm repository, a set of *P. spinosa* accessions from Sweden, and a group with apparently mixed identities that included “Krikon” plum and “Mirabelle sans nom” from INRA, a *P. spinosa* accession from Sweden, and two *P. cerasifera* replicates from UC Davis. *P. insititia* samples did not form a distinct clade and were scattered among autochthonous *P. domestica* and EUR germplasm. These results indicate a relatively close genetic similarity among all Eurasian plums regardless of ploidy level. The mixed groupings of the Eurasian plum accessions also indicates a high likelihood that at least some of the material is mis-identified or was subject to collection/processing errors. Interestingly, three *P. spinosa* genotypes from Portugal grouped with autochthonous *P. domestica* accessions “Wegierka wiedenska” and *P. insititia* “Tersen” from southern Sweden. These findings indicate the potential that at least some *P. domestica* and/or *P. insititia* accessions may represent ancestral germplasm, however, we could not definitively rule out the possibility that these accessions resulted from interspecific hybridization with *P. domestica*.

### Germplasm stratification—F_ST_ statistics and PCA analysis

We used Wright’s F-statistics (F_ST_) to explore the degree of differentiation between pomological groups and Eurasian plum species. F_ST_ estimates the level of reduction in heterozygosity when compared to the expected Hardy–Weinberg equilibrium^39^. Consistent with random mating, the inbreeding coefficient F_IS_ was low among *P. cerasifera* and negative within *P. domestica* pomological groups. The overall fixation index (F_ST_) for *P. domestica* groups was estimated to be 0.005–0.033 (Table 2) and, in agreement with small pairwise genetic distances, reflecting weak differentiation among the identified *P. domestica* clades.

**Table 2:** Number of private alleles and pairwise F_ST_ between plum pomological groups and species (interspecific hybrids and underrepresented species deleted)

	Private sites^a^	DAP	GRG	Pc	MIR	Psp	Pi
EUP	16232	0.018	0.006	0.044	0.005	0.026	0.009
DAP	3452		0.033	0.079	0.030	0.056	0.026
GRG	1990			0.086	0.023	0.054	0.021
Pc	3999				0.113	0.057	0.069
MIR	1186					0.058	0.018
Psp	12930						0.023
Pi	3269						

^a^17,339 SNPs in pairwise haplotype file

The F_ST_ values were not uniform across the dataset as differentiation between EUP and DAP was significantly stronger than between EUP and GRG or MIR groups; 0.018, 0.006 and 0.005, respectively. The highest differentiation was found between DAP and GRG and DAP and MIR (0.033 and 0.030, respectively). Overall the F_ST_ index was largely in the range of 0.05–0.15 for pairwise comparisons between DAP, GRG, MIR, or EUP vs. potential progenitor species *P. cerasifera* and *P. spinosa*. This range represents moderate to low differentiation according to Hartl and Clark^40^ indicating that *P. spinosa* is less differentiated from *P. domestica* groups than *P. cerasifera* (Table 2). The F_ST_ index for the EUP group vs. *P. spinosa* was significantly less than other pomological groups (0.026 vs. 0.05); suggesting that EUP is less diverged from the *P. spinosa* ancestor. This difference became more obvious after removing diploid *Prunus* species as well as the interspecific hybrids “Marianna 2634” and the *P. brigantina* × *P. cerasifera* hybrid from the dataset (Table S3).

Next, we conducted Principle Component Analysis (PCA) to estimate the genetic stratification of the germplasm. We compared the patterns of genetic variation using the entire dataset of 129,110 SNPs vs. filtered datasets generated with different minimum minor allelic frequencies (MAF) and missing rates (MAF = 1%, 3%, or 5% and missing rate = 10–30%) (data not shown). Optimal resolution of plum species and pomological groups was achieved at MAF = 5% and a call rate >80% (missing rate = 20%) resulting in an optimized set of 24,978 SNPs which were used for further genetic analysis.

**Fig. 4:**
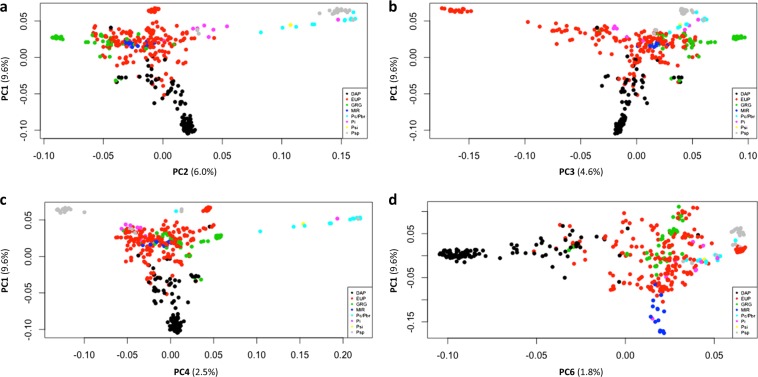
Principal component analysis of 405 plum accessions using 24,978 nuclear SNPs. Correlation matrixes calculated for first six PCs explain 26.8% of total variance: **a** PC1 vs. PC2; **b** PC1 vs. PC3; **c** PC1 vs. PC4; and **d** PC1 vs. PC6. Note: PC5 not shown. Color reflects different pomological groups and plum species: black- d’Agen prunes (DAP); red—European plums (EUP); green—Greengages (GRG); blue—mirabelles (MIR); cyan—*P. cerasifera*, *P. brigantina* and their hybrids (Pc/Pbr); magenta—*P. insititia* (Pi); yellow—*P. simonii* (Psi); gray—*P. spinosa* (Psp)

Principal components analysis (PCA) of the 24,978 SNP dataset identified 32 significant principal components (PCs) (explaining 43.72% of total variance). The first six PCs (9.6%, 6.0%, 4.6%, 2.5%, 2.3%, 1.8%—26.8% of variation in total) were selected for classifying/clustering accessions into groups (Fig. 4, PC5 not shown; Table S4).

A large proportion of the genetic variation remained unstructured. In agreement with pairwise F_ST_ statistics, DAP and potential progenitors of hexaploid plum, *P. cerasifera* and *P. spinosa*, were well differentiated along the first two principle components PC1 and PC2. Diploid *Prunus* species (*P. cerasifera* and *P. brigantina*) and *P. spinosa* were differentiated along PC4 (2.5% of variance). Plotting PC1 against PC3 and PC6 (4.6% and 1.8% of variance, respectively) gave only weak differentiation of GRG and MIR from EUP. This was in contrast to the relatively strong differentiation observed for DAP under all combinations.

### Discriminant analysis of pomological groups and progenitor’s species

**Fig. 5:**
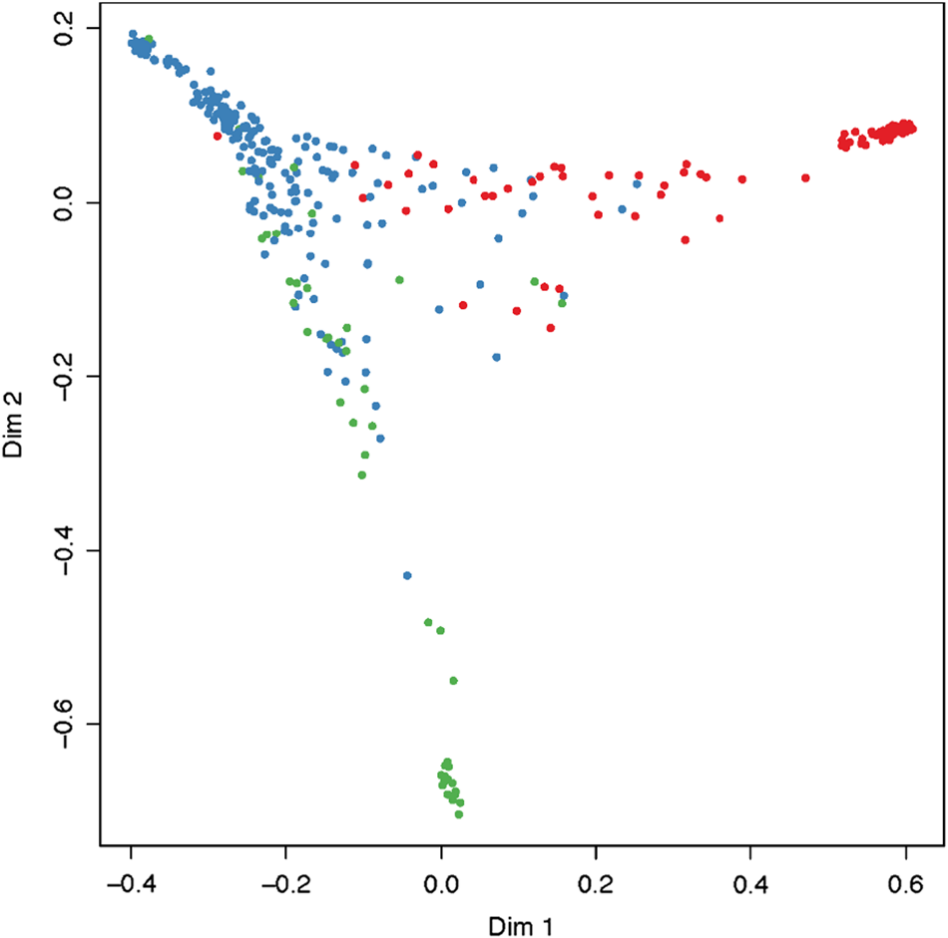
Multidimensional plot of the proximity matrix by randomForest analysis calculated using dataset of 405 plum accessions and 24,978 SNPs (ntree = 1000). Color indicates main plum groups used in analysis: green—greengages (GRG), red—d’Agen prunes (DAP), blue—remaining plum accessions germplasm (cPLM)

Random Forest (RF) is a machine-learning approach that allows classification of individuals into groups and the ranking of the relative importance of each SNP based on classification error. Each SNP is essentially removed one-by-one and the significance of its contribution evaluated. This approach was used to confirm whether the GRG and DAP groups can be effectively discriminated from the rest of the Eurasian plum germplasm. First, we applied the RF algorithm and constructed a multidimensional plot for the dataset composed only of three plum groups—DAP, GRG, and combined plums (cPLM) representing the rest of accessions, i.e., EUP, MIR, autochthonous plums, and related species (Fig. 5). We generated seven subsets of the most significant SNPs for separation of GRG and DAP from the rest of germplasm. The out-of-bag error (OOB) was in the range of 4.4–6.7%, supporting the differentiation of GRG and DAP. Orthogonal scattering of GRG and DAP likely reflected selection of different variables (SNPs) associated with breeding for different fruit types. This is in agreement with PCA results and F-statistics.

To extract a subset of the most significant genetic markers for differentiating these groups, we ran a RF analysis for DAP or GRG vs. the rest of the germplasm. After each round of calculations, we extracted the top 20 most significant SNPs and then combined them, retaining only unique marker names. This resulted in a list of 76 significant markers for DAP plums. In a similar manner, we generated a list of the 85 most significant markers for discriminating GRG from other plum groups. Next, we combined these SNPs and verified that they would reproduce the scattering pattern for GRG and DAP on a PCA plot (Figures S2, S3; Table S5). Lastly, Procrustes analysis was used to show the superimposed distributions of accessions for groups between the unreduced SNP set and the subset of 161 most informative SNPs (*P*-value < 0.001 by Monte Carlo permutation procedure, Figure S3). The results show that the subset of 161 markers may have prediction value for classification of plum germplasm by fruit type (GRG vs. DAP vs. EUP vs. MIR) as well as for possible genomic selection for fruit type in plum breeding programs. Next, we mapped the most significant SNPs against the peach genome (Fig. 1; Table S5). The SNPs were non-uniformly distributed on all eight peach pseudochromosomes, indicating that specific chromosomal regions may have been under selection in the breeding for GRG and DAP types (Fig. 1; Figure S4; Table S5). We analyzed the genes harboring these 161 SNPs to look for potential functional relationships (Table S4). The most abundant gene categories included sugar metabolism and transporters, cell wall modification enzymes, cell cycle regulation, RNA processing, and development.

### A rare cp marker is associated with d’Agen prunes

**Fig. 6:**
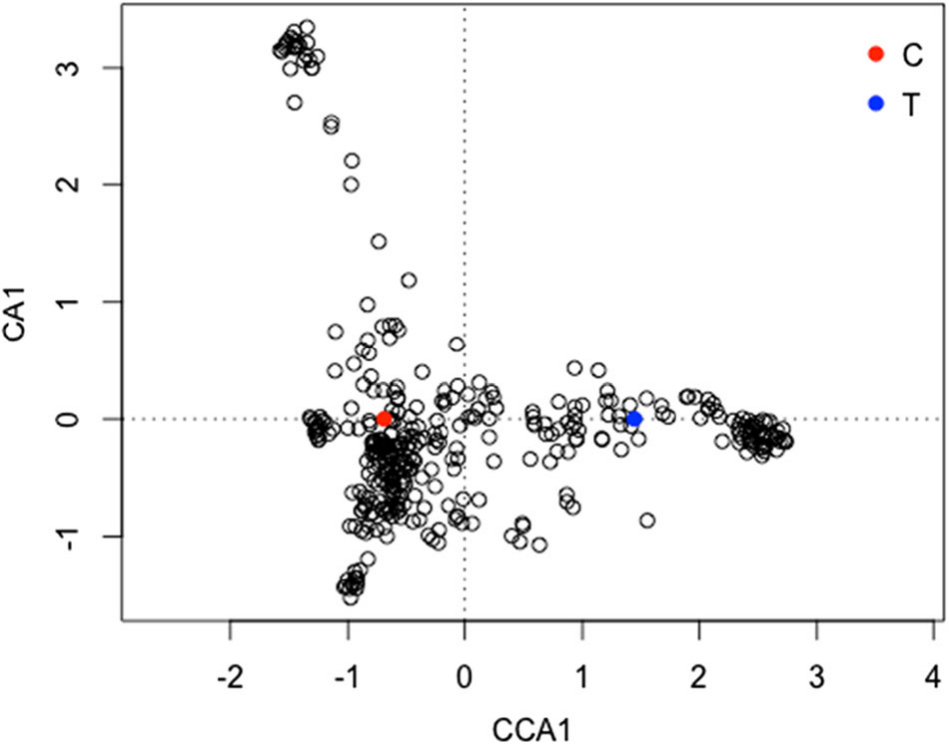
Canonical correspondence analysis (CCA) plot. Correlation matrix calculated using dataset of 371 plum accessions genotyped for 24,978 nuclear SNP markers (unconstrained axis). Constrained ordination was done along the CCA1 axis reflecting cp haplotypes C/T (no missing values allowed). *P* ≤ 0.001 (*n* = 999). Color indicated cp haplotype C (blue) and T (red)

Due to insufficient depth of reads, we failed to establish a cp haplotype for 34 accessions (8.40%), so these were removed from the dataset. In total, 371 samples were retained for analysis. A C/T substitution in the large subunit of the RUBISCO gene was found in 29.9% of the accessions, while 61.7% of cultivars had the cp haplotype C. Interestingly, the haplotype T was almost exclusively found in DAP, among which only 6.50% of samples had haplotype C. BLAST searches against available cp genomes in NCBI revealed that this residue is highly conserved among Angiosperms, suggesting the T haplotype is rare (data not shown). To uncover potential associations between nuclear markers and the cp haplotype we conducted Canonical Correspondence Analysis (CCA) using the vegan package in R^41^. The Permutation test (*P* < 0.001) confirmed a significant association with a subset of nuclear markers. These markers were scattered along the CCA1-axis, potentially reflecting their simultaneous introgression into plum germplasm along with the T haplotype (Fig. 6). In contrast, nuclear markers associated with the C haplotype were distributed along the CA1-axis in an unconstrained fashion, indicating its association with two or more different sources of nuclear markers. The distribution of this cp haplotype mimics the strong PC differentiation observed for DAP relative to GRG, MIR, and EUP (Figure S5).

## Discussion

### Differentiation of *P. domestica* pomological groups

Overlapping distribution of morphological traits among pomological groups of *P. domestica* as well as among seedlings derived from self-pollinated cultivars prompted some early authors to propose that subdivision of *P. domestica* cannot be justified from a genetic point of view^42,43^. Based on the analysis of worldwide germplasm presented here, we report clear genetic evidence that at least some pomological groups of *P. domestica* have distinctive genotypic signatures that can be used for assignment of unknown accessions and paves the way for future genome-enabled prediction of agronomic traits in breeding material.

Two pomological groups, DAP and GRG, were clearly separated from each other and from the rest of the *P. domestica* germplasm. GRG and DAP formed individual clades on the dendrogram derived from the complete dataset of 129,110 polymorphic SNPs and were readily separated by PCA analysis using a representative dataset of 24,498 SNPs. Accordingly, the highest fixation index between all pomological groups (F_ST_ >  0.03) indicated that DAP and GRG are the most differentiated among Eurasian plum germplasm. The relatively strong differentiation of DAP and GRG was also supported by RF analysis, an advanced machine-learning approach^44^. Recently, successful implementation of RF for site-by-site classification of Atlantic and Chinook salmon species allowed the establishment of a relevant, non-redundant and maximally reduced panel of genetic markers^45^. Identification of informative DNA markers useful for differentiating populations was also achieved in cattle breeds^46^ and perennial ryegrass^47^. This report provides support for the efficiency of RF in analyzing and unmasking NGS datasets.

We were able to computationally identify two subsets of SNPs that were most informative for assigning unknown accessions to the GRG and DAP groups (subsets 76 and 85, respectively). Based on the uneven distribution of these SNPs across the genome, we hypothesize that these genomic regions may harbor important genes for fruit type or other agronomic traits that are distinct between the DAP and GRG groups. Historically, these pomological groups were selected for different fruit usage such as drying vs. fresh consumption. In many cases these SNPs were within or proximal to orthologues of proteins known to have key roles in sugar metabolism and transport which could underlie important fruit quality differences. Thus, these SNPs may serve as good markers for screening hybrid material in breeding programs aimed at dried or fresh types of fruits.

Despite the ability to clearly resolve some *P. domestica* pomological groups, the results revealed relatively small genetic distances between them. Thus, *P. domestica* was likely derived from a limited set of founders where breeding consisted primarily of self-pollination and/or hybridization among selected siblings. This conclusion was made possible by the inclusion of siblings derived from a self-pollinated “Improved French” individual. This also allowed us to estimate branch length cutoffs to distinguish clonal accessions from siblings. Clonal subclades were identified within all pomological groups and Eurasian plum accessions, consistent with the historical practice of making clonal selections from vegetative buds or sports. Importantly, nearly all commercially grown DAP varieties from France, USA, Argentina, and Australia were found to be clonal, establishing that the plum industry worldwide is predominantly a monoculture. A surprising finding was the unique cp haplotype T that prevailed in DAP plums but was not detected in related *Prunus* species. This suggests that DAP types were historically not cross hybridized to EUP, GRG, or MIR plums and were derived from a distinct hybridization event. Interestingly, DAP plums are very old varieties as they date to before the 12th century where they were reportedly brought to France directly from Syria^37^. Thus, their differentiation from other *P. domestica* cultivars despite the relatively short genetic distances between them raises the possibility that this cp haplotype is a remnant of one of the inter-specific events that gave rise to *P. domestica* in the Caucasus. Given the lack of evidence for this haplotype in all Eurasian accessions tested, it is possibly derived from a theoretical 3rd Eurasian genomic contributor. Such a possibility is supported by CCA analysis that found sets of nuclear markers were associated with the cp T haplotype. Future genome sequencing of *P. domestica* and its progenitors will be necessary to confirm these hypotheses.

The sequence-based genotyping method used here has limitations caused by the lack of a plum reference assembly and the reliance on the *P. persica* genome for reference assembly of the *P. domestica* sequences. As shown for Sugarcane and Saccharum Complex, the quality of the reference assembly strongly influences GBS performance in polyploids^48^. Consequently, we likely missed an opportunity for discovery of polymorphic markers derived from intergenic regions, as well as SNPs that can discriminate between subgenomes (homoeoSNPs). Despite this limitation, the results of our analyses were highly consistent with known genetic relationships and support the robustness of the SNP calling pipeline.

### Relationship of *Prunus domestica* to other Eurasian plums

Questions regarding the origin of *P. domestica* have been studied and debated for over a century. The wide range of phenotypic diversity observed in *P. domestica* and its Eurasian relatives along with their overlapping distributions has made resolving these questions particularly difficult. A handful of competing theories have been proposed that are not all mutually exclusive: [1] *P. domestica* is entirely of *P. cerasifera* origin and represents an autopolyploid event of this species, [2] *P. domestica* is a hexaploid of *P. spinosa* which is a discrete species, [3] *P. domestica* is an interspecific hybrid between a diploid *P. cerasifera* and a tetraploid *P. spinosa*, and [4] *P. domestica* is a hybrid of interspecific hybrids having a hexaploid chromosome complement composed of *P. cerasifera*, *P. spinosa*, and possibly contributions from other Eurasian plum species. The results presented here all but rule out models [1] and [2] but do not definitively resolve between [3] and [4]. Consistent with the prevailing theory proposed by early researchers, our data are most consistent with model [4] whereby one subgenome was contributed by diploid *P. cerasifera* and the other from a tetraploid *P. spinosa* that itself is an interspecific hybrid between diploid *P. cerasifera* and a second, as of yet, unknown *Prunus* species such as *Prunus ramburii* Boiss as suggested by Reales et al. in 2010^6,20,32^. The endemic species *P. ramburii* was not included in our study but mixed grouping of *P. brigantina* and its hybrid *P. brigantina × P. cerasifera* with *P. spinosa* and *P. cerasifera* accessions agrees with results reported by these authors and Shi et al. in 2013^6,20,32,49^. Likewise, the lack of the DAP cp haplotype in the *P. cerasifera* or *P. spinosa* accessions tested imply that this haplotype may be derived from a distinct ancestral species. Irrespective of whether a 3rd species contributed to the *P. domestica* genome, the relatively close genetic distances among the germplasm supports the hypothesis by Eryomine, 1990 that some or all of the interspecific hybridization events that gave rise to *P. domestica* did not occur naturally but were artificially selected for by early Eurasian societies^15^. Such a scenario is consistent with the lack of known wild *P. domestica*, the low genetic diversity across the germplasm reflecting a more recent origin, and the low pollination and fertility rates among experimental interspecific hybrids which would presumably have had tremendous difficulty naturally establishing themselves^15,50^. In addition, historical evidence suggesting widespread simultaneous cultivation of *P. domestica*, *P. cerasifera* and *P. spinosa* is consistent with the current state of wild Eurasian germplasm that appears to be comprised by feral populations with relatively low genetic diversity. Whole genome sequencing, accompanied with additional genotyping using a broader range of Eurasian plum germplasm will be necessary to fully resolve these possibilities.

## Materials and methods

### Plant material

Plant material for this study included samples from 251 cultivars and forms of *P. domestica* held at the germplasm repository at INRA (Bordeaux, France), 66 cultivars from germplasm collections maintained at University of California at Davis and two USDA Agricultural Research Service (ARS) sites—National Clonal Germplasm Repository (NCGR) and the Appalachian Fruit Research Station (AFRS, Kearneysville, WV). Also, 31 accessions representing related Eurasian plum species and cultivated varieties were obtained from Argentina, Australia, Portugal, Sweden, and Ukraine. A complete list of accessions, their origin and pomological characteristics is provided in Supplemental Materials (Table S1). Classification for pomological groups was conducted according to Neumüller^12^ who separated plums, prunes, Reine Claudes (Greengages), Mirabelles, primitive forms and autochthonous forms. Finally, 34 genotypes derived from self-pollination of the cultivar “Improved French” maintained at AFRS (Kearneysville, WV) were used to aid in distinguishing self-pollinated genotypes from clonal material.

To verify the reproducibility of genotyping and dendogram construction, we randomly selected and sequenced 23 of the DNA samples twice. Technical replicates were included in analyses under cultivar names followed by “.1” and “.2”. Sixteen sets of biological replicates were also included, consisting of independently extracted DNAs from the same cultivar present in different locations, and listed under cultivar names followed by numbers 1 or 2 without a period.

### DNA extraction, library construction, and sequencing

Genomic DNAs were isolated using either the Qiagen DNeasy DNA extraction kit (Qiagen, Inc.) following manufacturer’s instruction or a modified CTAB protocol by Kubisiak et al.^51^. the quality and integrity of the DNA was examined using a NanoDrop ND-8000 (Thermo Fisher Scientific Inc., USA) followed by electrophoresis on 1% agarose gels. Quantification of DNA samples was done using QuantiFluor® dsDNA kit (Promega, Inc.) and a Synergy H1 microplate reader (Biotek, USA).

Libraries for sequencing were constructed following protocols by Elshire et al.^52^ with a few modifications. Briefly, 100 ng of genomic DNAs were digested in the presence of *Pst*I (New England Biolabs, Inc.). Custom barcoded adapters were ligated to the *Pst*I-compatible overhang, and samples were pooled and then purified with a commercial PCR purification kit (Qiagen, Inc), and finally quantified with a NanoDrop ND-1000. For test-amplification, 46 ng of DNA was amplified with common primers and NEBNext® High-Fidelity 2X PCR Master Mix (New England Biolabs, Inc.) as outlined in Elshire et al.^52^. The total volume of the PCR mix (50 µl) was divided into five tubes (10 µl each). Typically, 12, 14, 16, and 18 cycles were set up for testing against a non-amplified control. DNA fragments were resolved on 1.5% agarose gels against the Hi-Lo™ DNA marker (Bionexus, USA) to select optimal number of cycles for fragment enrichment in the range of 200–700 bp. Final libraries for genotyping-by-Sequencing (GBS) were amplified in two replicates (combined volume 100 µl) and purified using Mag-Bind^®^ Total Pure NGS magnetic beads (Omega Bio-Tek, GA, USA). Two-step size selection with 0.4× and 0.8× volumes of magnetic beads depleted fragments larger than 1.5 kb and smaller than 121 bp, respectively. Quality checks for GBS libraries were done using a 2100 BioAnalyzer (Agilent Technologies, CA, USA). Fragment size distribution agreed with that predicted from “in silico” digestion of the *P. persica* reference genome with the *Pst*1 restriction enzyme (not shown). Amplified GBS libraries were quantified using a Qubit^®^ 2.0 fluorometer (ThermoFisher Scientific, Inc.) and were submitted for sequencing. Four 96-plexed and one 48-plexed sets of libraries were pair-end sequenced (2 × 125 bp) on a single lane each using the Illumina HiSeq 2500 instrument at the core facilities of the Medical University of South Carolina (Charleston, SC). Raw reads for all 405 accessions were deposited into NCBI’s SRA database (BioProject PRJNA436025, SRA accession: SRP134093).

### Raw sequence data processing and genotyping

Data processing and SNP genotyping were performed using Stacks v.1.44-v.1.45^39,53^. Briefly, raw paired-end (PE) sequences were de-multiplexed according to barcodes, trimmed to remove adapters and low quality (<phred33) sequences, and filtered for the presence of the *Pst*I restriction sites. Typical success rate was in a range of 95–97%. Using the GSNAP software package^54^ reads were aligned to the *P. persica* v 2.1 genome^55,56^. An inclusive catalog of tags and SNP genotypes (bi-allelic sites only) were generated using the “ref_map.pl” command with default parameters. The SNP genotypes were generated in the population mode of Stacks, recorded in the Variant Call Format (VCF) and filtered using VCFtools^57^. To simplify analyses and limit errantly called variants, all indels and multi-nucleotide variants were deleted, leaving only bi-allelic SNPs and invariant sites. Individual genotypes were filtered to remove calls supported by less than 5 reads. Only SNPs present in more than 80% of all accessions (330 out of 405) were kept in the dataset for genetic analyses.

Structural and functional annotation of SNPs in coding and non-coding regions on the *P. persica* pseudochromosomes was done using the SnpEff 4.3e (build 2016-11-19) variant annotation and effect prediction tool^58^. The physical positions (bp) of reference-based SNPs were correlated with the GFF file of the peach genome annotation v2.1 deposited at the Genome Database for Rosaceae (GDR)^56,59^. The genomic distributions of SNPs across all eight peach chromosomes (pseudomolecules) were plotted individually based on their physical positions (100K screening window) and visualized using the Circos visualization tool^60^.

The same analytical pipeline was used for isolation of genomic fragments derived from the peach chloroplast (cp) genome. Demultiplexed reads were aligned against the *P. persica* cp sequence deposited at GenBank (HQ336405.1). The cp-derived polymorphic sites were called using “ref_map” command adjusted for ploidy level 1^39,53^.

### Genetic diversity and population structure analysis

The SNPRelate package in R^61^ was used to investigate genetic diversity of the plum germplasm. A distance matrix derived from the Euclidian metric was calculated and used in hierarchical cluster analysis. A dendrogram was constructed using the between-group average linkage method, or UPGMA. The confidence interval of the genetic relationships among the accessions was determined by performing 5000 bootstrap replicates. A graphical representation of the resulting tree (in Newick format) was visualized in Geneious R10^35^. Wright’s fixation index (F_ST_) was used to explore the degree of differentiation between pomological groups and species. F_ST_ values and standard errors for all pairwise comparisons were calculated using a “population” mode in Stacks^39^.

Principal component analysis (PCA) was performed using the R package SNPRelate^61^. The VCF file with genotypic data was converted into the genomic data structure (GDS) file format, filtered for MAF ≥ 0.05 and missing rate ≤0.20 and used for PCA analysis with the snpgdsPCA function. The percentage of explained variation was calculated for the first six PCs used for plotting the genotypes on a two-dimensional scale. All plots were generated using ggplot2 included with the library.

The nonparametric tree-based ensemble random forest (RF) analysis^44^ was executed using the R package Random Forest with 1000 permutation trees^62^. The variable (i.e., SNP) importance was estimate based on the Mean Decrease Accuracy (MDA) metric that measures contribution of individual SNPs to an accuracy of prediction when all other SNPs are constant. Higher MDA values indicate large changes to the prediction power, and hence, the importance of the SNP. In total, 7 output datasets of the most significant SNPs were generated with the “ntree = ” parameter settings of 250, 500, 1000, 2000, 5000, 8000, and 15,000. The distribution of accessions on a PCA plot (with most significant SNPs) and RF plot was compared using the Procrustes technique implemented in the R package vegan 2.4-4^41^. Association between nuclear markers and cp haplotypes was estimated by the CCA using vegan in R. Significance of constrain was estimated with the *anova.cca* function by permutation test under reduced model (*n* = 999).

## Electronic supplementary material

Supplemental materials

Figure S1

Figure S2

Figure S3

Figure S4

Figure S5

Table S1

Table S2

Table S3

Table S4

Table S5
