# Supplementary material for: Genetic characterization of worldwide Prunus domestica (plum) germplasm using sequence-based genotyping
Source: Hortic Res. 2019 Jan 1;6:12. doi: 10.1038/s41438-018-0090-6 (PMC6312543; doi:10.1038/s41438-018-0090-6)
Supplement: Supplementary file 1 — Supplemental materials [file 41438_2018_90_MOESM1_ESM.docx]

**Supplemental files**

Figure S1. Genetic similarity within plum germplasm. Dendrogram was generated using UPGMA clustering method on dissimilarity matrix computed for 405 accessions and 103,382 nuclear markers (high-resolution image). Branches - in ascending order based on pairwise distances. Different *Prunus* species and pomological groups are defined by colors –*P. cerasifera* and tetraploid *P. spinosa* (blue), European plums (black), mirabelles (brown and amber), greengages (green and bold green) and d’Agen prunes (fuchsia and lilac). More intensive colors are given to founding clonal varieties with typical morphological traits.

Figure S2. PCA plot of 405 accessions based on 161 most significant SNPs for discrimination of DAP (red) and GRG (green) plums from the rest of germplasm (cPGM, black).

Figure S3. Procrustes-transformed plot of the first two principal components of genetic variation of 405 plum accessions on multidimensional plot generated by randomForest (24,278 SNPs) and PCA plot generated from 161 most significant SNPs selected by randomForest. Grey dots – MDS plot by randomForest. Colored dots - PCA plot with 161 most significant SNPs: black – cPLM, red – DAP; green – GRG).

Figure S4. Genomic regions most strongly associated with the differentiation of DAP (red) vs GRG (green) plum types. Chromosomal segments are visualized on the peach genome as a reference.

Figure S5. PCA analysis of 405 accessions based on 24,978 SNPs. Accessions are colored according to cp haplotype.

Table S1. Plum accessions genotyped in this study including sample information, assignment to pomological groups, and cp haplotypes.

Table S2. cp genotype data file in VCF format.

Table S3. Matrix of pairwise distances between plum accessions used for dendrogram construction.

Table S4. PCA variance of six first PCs along peach reference pseudochromosomes.

Table S5. Structural and functional annotation most significant SNPs selected by randomForest for classification greengages (GRG) and d'Agen prunes (DAP).
