# Supplementary figures and images for: Genetic characterization of worldwide Prunus domestica (plum) germplasm using sequence-based genotyping
Source: Hortic Res. 2019 Jan 1;6:12. doi: 10.1038/s41438-018-0090-6 (PMC6312543; doi:10.1038/s41438-018-0090-6)

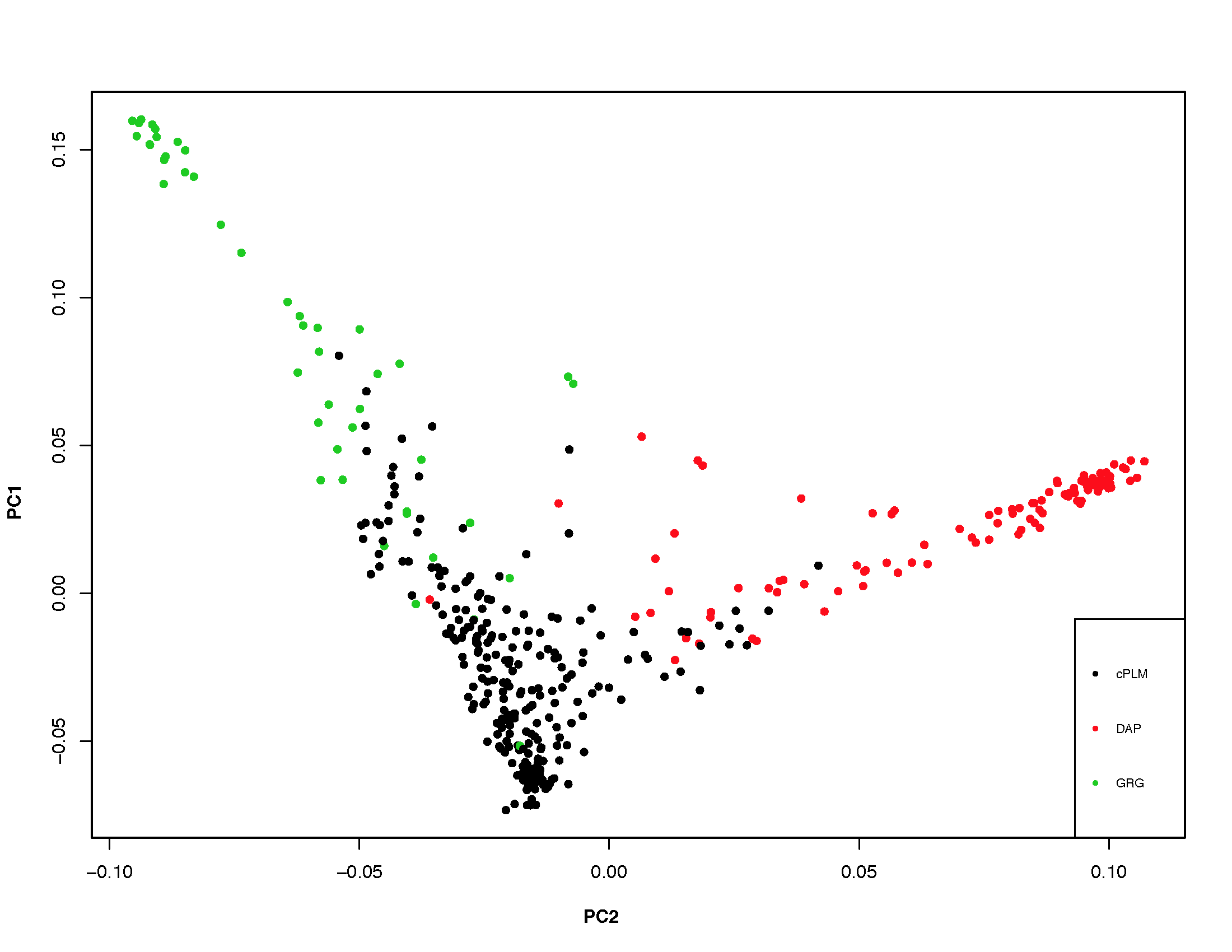

Supplement: Supplementary file 3 — Figure S2 [file 41438_2018_90_MOESM3_ESM.tif]

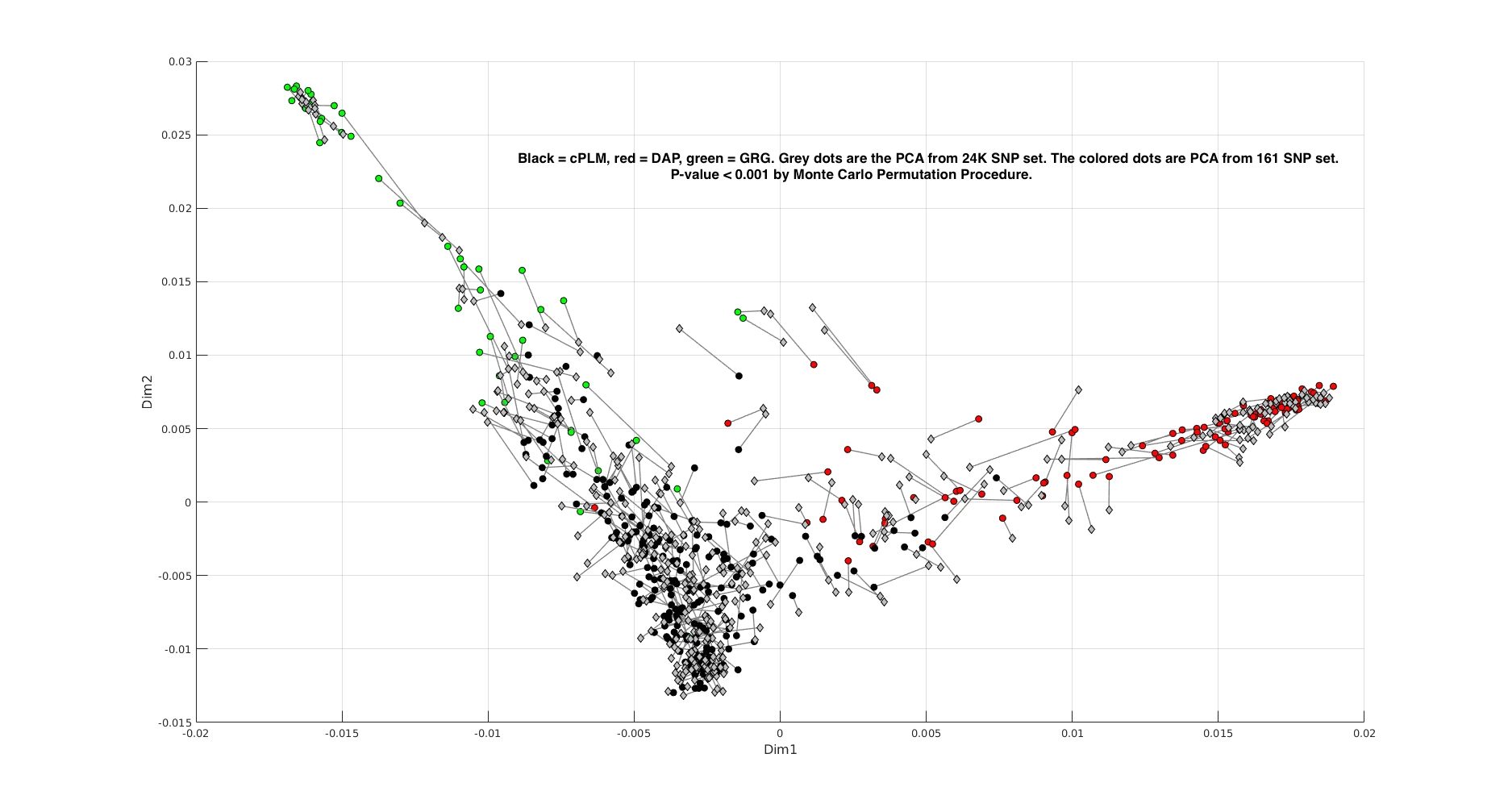

Supplement: Supplementary file 4 — Figure S3 [file 41438_2018_90_MOESM4_ESM.tif]

Figure S4

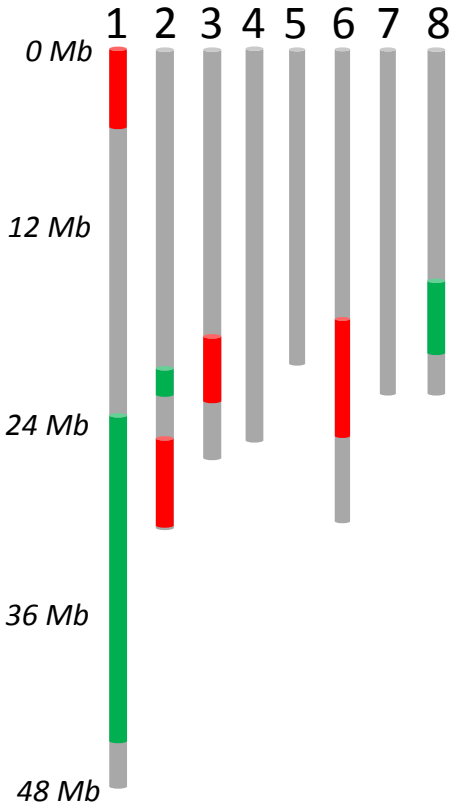

Supplement: Supplementary file 5 — Figure S4 [file 41438_2018_90_MOESM5_ESM.pdf]

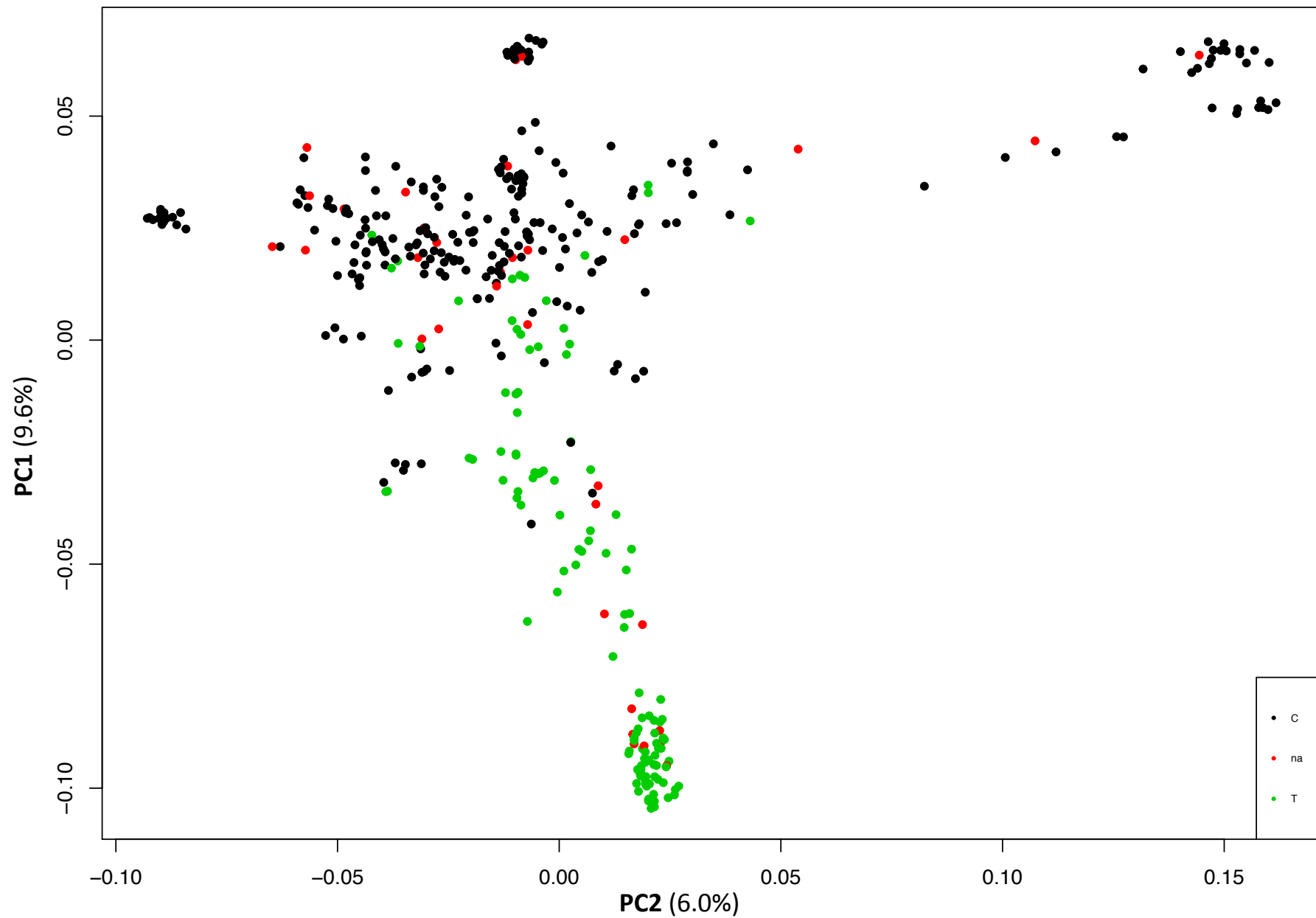

Supplement: Supplementary file 6 — Figure S5 [file 41438_2018_90_MOESM6_ESM.pdf]
