# Supplementary material for: Genetic characterization of worldwide Prunus domestica (plum) germplasm using sequence-based genotyping
Source: Hortic Res. 2019 Jan 1;6:12. doi: 10.1038/s41438-018-0090-6 (PMC6312543; doi:10.1038/s41438-018-0090-6)

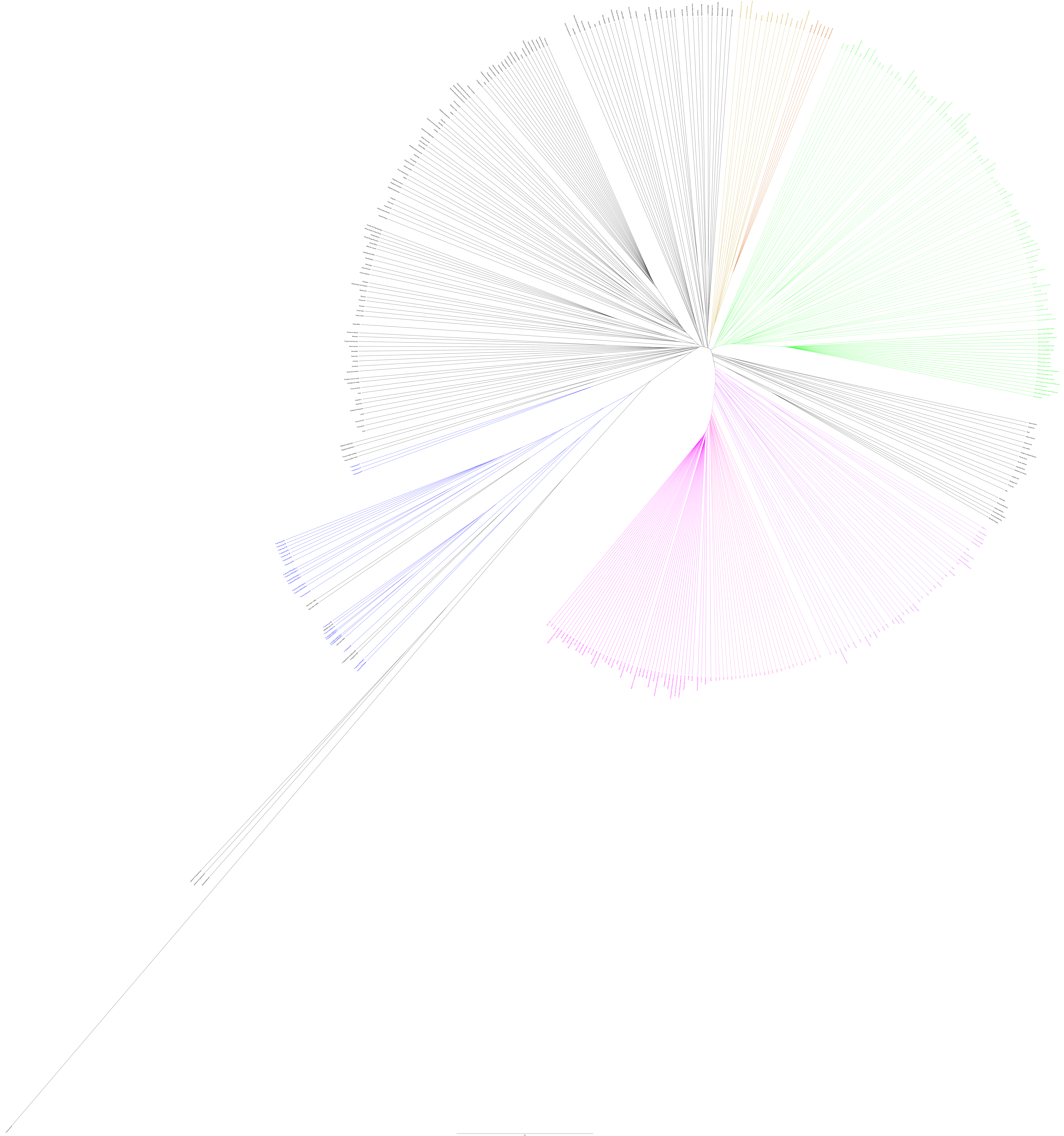

Supplement: Supplementary file 2 — Figure S1 [file 41438_2018_90_MOESM2_ESM.pdf]
